# Interleukin (IL)-13 promoter polymorphisms (-7402 T/G and -4729G/A) condition susceptibility to pediatric severe malarial anemia but not circulating IL-13 levels

**DOI:** 10.1186/1471-2172-14-15

**Published:** 2013-03-25

**Authors:** Winnie A Okeyo, Elly O Munde, Wilson Okumu, Evans Raballah, Samuel B Anyona, John M Vulule, John M Ong’echa, Douglas J Perkins, Collins Ouma

**Affiliations:** 1 Department of Biomedical Sciences and Technology, School of Public Health and Community Development, Maseno University, Maseno, Kenya; 2 Department of Medical Biochemistry, School of Medicine, Maseno University, Maseno, Kenya; 3 Department of Medical Laboratory Sciences, School of Health Sciences, Masinde Muliro University of Science and Technology, Kakamega, Kenya; 4 Center for Global Health Research, Kenya Medical Research Institute, Kisumu, Kenya; 5 University of New Mexico/KEMRI Laboratories of Parasitic and Viral Diseases, Centre for Global Health Research, Kenya Medical Research Institute, Kisumu, Kenya; 6 Center for Global Health, Department of Internal Medicine, University of New Mexico, Health Sciences Centre, Albuquerque, NM, USA; 7 Directorate of Research, Publications and Consultancies, Maseno University, Kisumu-Busia Road, Private Bag, Maseno, Kenya

**Keywords:** IL-13, Promoter polymorphisms, Haplotypes, Severe malaria anemia

## Abstract

In holoendemic *Plasmodium falciparum* transmission areas such as western Kenya, severe malarial anemia [SMA, hemoglobin (Hb) < 6.0 g/dL, with any density parasitemia] is the most common clinical manifestation of severe malaria resulting in high rates of pediatric morbidity and mortality in these regions. Previous studies associated interleukin (IL)-13 with pathogenesis of different infectious diseases, including *P. falciparum* malaria. However, the functional roles of polymorphic variants within the IL-13 promoter in conditioning susceptibility to SMA remain largely unexplored. As such, the association between the IL-13 variants -7402 T/G (rs7719175) and -4729G/A (rs3091307) and susceptibility to SMA was determined in children (n = 387) presenting with clinical symptoms of falciparum malaria and resident in a holoendemic transmission region in western Kenya. Our results indicated no difference in the proportions of individual genotypes among children presenting with non-SMA (n = 222) versus SMA (n = 165). Similarly, there was no associations between the individual genotypes (-7402 T/G and -4729G/A) and SMA. Additional analyses, however, revealed that proportions of individuals with -7402 T/-4729A (TA) haplotype was significantly higher in children presenting with SMA than non-SMA group (*P* = 0.043). A further multivariate logistic regression analyses, controlling for confounding factors, demonstrated that carriage of the TA haplotype was associated with increased susceptibility to SMA (OR; 1.564, 95% CI; 1.023-2.389, *P* = 0.039). In addition, circulating levels of IL-13 were comparable between the clinical groups as well as across genotypes and haplotypes. Collectively, findings presented here suggest that haplotypes within the IL-13 promoter at -7402 T/G and -4729G/A may modulate SMA pathogenesis, but do not affect circulating IL-13 levels.

## Background

Plasmodium malaria is one of the leading causes of morbidity and mortality in the world [1]. World Health Organization (WHO) estimated that 216 million malaria episodes occurred in the year 2010, resulting in approximately 655,000 deaths, of which 91% occurred in the sub-Saharan Africa [2]. Children under five years of age bear the greatest disease burden, with a global malaria related mortality of 86% documented in 2010 [2]. In malaria endemic regions of sub-Saharan Africa, greater than 90% of malaria-related deaths are attributable to *Plasmodium falciparum*, the most virulent of the five malaria parasite species known to cause human malaria [3]. The clinical spectrum of severe falciparum malaria often ranges from mild symptoms to life threatening complications which include, but are not limited to, severe anemia, respiratory distress, cerebral malaria (CM), hyperparasitemia, metabolic acidosis, kidney failure, and hypoglycemia [4]. In western Kenya, a falciparum malaria holoendemic region, the most common clinical manifestation of severe malaria is severe malarial anemia [SMA (Hb < 6.0 g/dL)], with any density parasitemia [5,6], with respiratory distress, hypoglycemia and CM reported only in rare cases [7-9]. In addition, the etiology of SMA is complicated by the presence of co-infections and by host genetic factors [9-14]. Furthermore, additional studies have demonstrated that immunity against SMA is characterized by dysregulation of innate types 1 and 2 inflammatory mediators, mainly cytokines, chemokines and effector molecules [15-17].

Among the inflammatory mediators implicated in malaria pathogenesis is interleukin (IL)-13, a type 2 cytokine that shares structural and functional characteristics with IL-4 [18,19]. IL-13 is a pleiotropic cytokine predominantly produced by activated T lymphocytes, mast cells, basophils, natural killer (NK) cells and dendritic cells [20]. It functions as an immune modulator on a wide range of cell types, including macrophages, NK cells, fibroblasts, eosinophils, airway smooth muscle cells, and endothelial cells [20]. Elevated levels of IL-13 have been implicated in allergic responses and in parasitic infections [21-23]. Recently, IL-13 has emerged as a negative predictor of hemoglobin (Hb) levels for pediatric malarial anemia [17].

The human IL-13 gene has been mapped to the 5q31-q33 region, located on chromosome 5, which contains additional genes encoding cytokines, including IL-3, IL-4, IL-5, IL-9, and IL-12B [24-28]. Polymorphisms in the promoters of genes coding for these cytokines have been shown to alter susceptibility to infectious diseases [11,25,29,30]. Several polymorphic variants identified in the IL-13 gene region [25,30,31] have been associated with various diseases including allergic rhinitis [32], asthma [33], atopic dermatitis [22], and schistosomiasis [25]. In addition, studies in Thai adults demonstrated that a single nucleotide polymorphism (SNP) in the IL-13 promoter (IL-13 -1055C > T) is associated with severe malaria [31]. However, to date, no studies have reported the associations between IL-13 promoter variants -7402G/T (rs7719175) and -4729G/A- (rs3091307), their haplotype constructs and SMA in pediatric populations resident in *P. falcipuram* holoendemic transmission region such as in western Kenya. The IL-13 promoter variants -7402G/T (rs7719175) and -4729G/A- (rs3091307) were selected based on the mutant allele frequency ~10% in reference African Yoruba population (http://hapmap.ncbi.nlm.nih.gov/downloads/frequencies/). Consequently, the current study investigated the associations between two IL-13 promoter variants, haplotypes and susceptibility to SMA in a phenotypically well-defined cohort of children aged 3–36 months presenting with clinical symptoms of malaria in a rural setting of western Kenya.

## Methods

### Study site

The study was carried out in Siaya District Hospital (SDH), a *P. falciparum* holoendemic transmission region in western Kenya. Residents in this region are predominantly of the Luo ethnic group (>96%), thus providing a homogeneous population suitable for a genetic-based study [5]. Malaria accounts for the highest number of childhood morbidities and mortalities in this area [6,17]. The major malaria vectors are *An. gambiae s.s*., *An. arabiensis* and *An. funestus*[34].

### Study participants

The study recruited parasitemic patients (n = 387), who presented with clinical symptoms of malaria at SDH. Both male and female children aged between 3 and 36 months visiting SDH for the first time with symptoms of malaria qualified for enrollment into the study. Informed consent to participate in the study was obtained from the parents/legal guardians of the children. Questionnaires were used to collect relevant geographical and clinical information. Any patient who had been hospitalized before in their lifetime, or who had received anti-malarial treatment two weeks prior to enrollment was excluded from the study. Children with CM, though very rare, and children with non-falciparum malaria were also excluded from the study. Parasitemic children with Hb levels of >6.0 g/dL were classified as non-severe malarial anemia (non-SMA) while those with Hb <6.0 g/dL were classified as having SMA. This classification was based on a previous large-scale longitudinal study that examined the distribution of >14,000 Hb measurements in an age- and geographically-matched reference population in western Kenya [35]. The study approval was obtained from the Ethics Review Committees of the Kenya Medical Research Institute (KEMRI).

### Sample processing

For children who met the inclusion criteria, blood samples from heel or finger pricks (<100μL) were obtained and used to determine parasite density by preparation of thick and thin blood smears stained using Giemsa reagent. In addition, venous blood, approximately 3 mL, was collected into EDTA-containing vacutainer tubes. About 100 μL of blood was bloated onto FTA Classic cards for use in DNA extraction for genetic analyses. A complete blood count was carried out using a Beckman Coulter AcT Diff2 (Beckman-Coulter Inc., Miami, FL) for hematological parameters including Hb levels, red blood cell (RBC) counts, hematocrit and mean corpuscular volume. Plasma was separated from the remaining blood, which was snap-frozen at -80 °C until use. Bacterial co-infections were investigated by incubation of blood collected in trypticase soy broth with sodium polyanethanol sulfonate (SPS) for 18 hours and bacteria identified by gram staining procedures, colonial characteristics, appearances and biochemical and serological tests. Sickle-cell status was determined by alkaline cellulose acetate electrophoresis while G6PD deficiency was determined by a fluorescent spot test using glucose-6-phosphate and oxidized NADP (NADP+) where samples were scored as normal (high emission), intermediate (moderate emission), or deficient (no emission).

### DNA extraction

DNA was extracted from the FTA Classic card and filter papers using the Gentra Systems DNA extraction protocol (Gentra Systems, Minneapollis, MN, USA). To confirm the presence of DNA, a house-keeping gene, human glucose-3-phosphate dehydrogenase (hG3PDH) was amplified.

### IL-13 genotyping

Applied Biosystems SNP-specific primer-probe combinations (TaqMan^®^ SNP Genotyping Assays, assay ID; C_31237837_10 and C_27452177_20) were used to genotype IL-13 -7402 T/G and -4729G/A SNPs, respectively, on the Applied Biosystems StepOnePlus™ Real-Time PCR System (Applied Biosystems, Foster City, CA, USA), according to the manufacturer’s instructions.

### Cytokine measurements

Plasma IL-13 levels were measured using the enzyme-linked immunosorbent assay (cytokine-ELISA), on an ELx800™ Absorbance Microplate Reader (BioTek^®^ Instruments, Inc. Winooski, VT, USA), according to the manufacturer’s specifications for IL-13 detection and levels computed using the Gen5 software (BioTek^®^ Instruments, Inc. Winooski, VT, USA). The detection limit for IL-13 was 10 pg/mL.

### Data management and analysis

Data analyses were performed using SPSS^®^ statistical software package version 19.0 (IBM SPSS Inc., Chicago, IL, USA). The IL-13 (-7402 T/G and -4729G/A) promoter haplotypes were constructed using HPlus software program (Version 2.5). Comparison of proportions between and within groups was computed using chi-square (*χ*^2^). Comparison of cytokine and Hb levels across individual IL-13 promoter variants was performed using Kruskal-Wallis test while pairwise group comparisons was by Mann–Whitney *U* test. Association between genotypes/haplotypes and SMA was determined using the multivariate logistic regression analyses models, controlling for potential confounders including age, gender, bacteremia, sickle cell trait (HbAS) and glucose-6-phosphate dehydrogenase (G6PD) deficiency. Statistical significance was considered at *P* ≤ 0.050.

## Results

### Demographic, clinical, and laboratory characteristics of the study participants

To determine the role of the IL-13 promoter variants (-7402 T/G and -4729G/A) in conditioning susceptibility to SMA, children (n = 387), aged 3–36 months presenting with *P. falciparum* malaria were categorized into two groups according Hb levels as non-severe malarial anemia (non-SMA, Hb ≥ 6.0 g/dL, any density parasitemia; n = 222) and severe malarial anemia (SMA, Hb < 6.0 g/dL, any density parasitemia; n = 165). The demographic, clinical, and laboratory characteristics of the study participants are summarized in Table 1. Proportions of males versus females in the clinical groups were comparable *(P* = 0.862). In addition, IL-13 levels (*P* = 0.415) and parasite density *(P* = 0.530) were also comparable between the clinical groups. Further analyses revealed that children in the non-SMA group were significantly older than those in the SMA group (*P* < 0.001). As expected based on stratification, the hematological parameters, among them Hb, hematocrit, red blood cell (RBC) count, red blood cell distribution width (RDW) and mean corpuscular hemoglobin concentration (MCHC) were all significantly different between the SMA and non-SMA groups (*P* < 0.001 in all cases).

**Table 1:** Clinical, demographic and laboratory characteristics of the study participants

**Characteristics**	**non-SMA (Hb ≥ 6.0 g/dL)**	**SMA (Hb < 6.0 g/dL)**	***P *value**
**No. of participants (n = 387)**	**222**	**165**	
Gender, n (%)			
Male	115 (51.8)	84 (50.9)	0.862^a^
Female	107 (48.2)	81 (49.1)	
Age, months	11.0 (10.0)	8.0 (8.0)	**<0.001**^**b**^
IL-13 concentration (pg/mL)	31.6(26.5)	35.4(38.07)	0.415^b^
Parasites/μL	29960.1(40957.8)	20639.1(42359.6)	0.530^b^
**Hematological Parameters**			
Hemoglobin, g/dL	8.0 (3.0)	4.9 (1.0)	**<0.001**^**b**^
Hematocrit,%	24.8 (7.3)	15.8 (5.0)	**<0.001**^**b**^
RBCs, × 10^12^/μL	3.7 (1.2)	2.2 (0.9)	**<0.001**^**b**^
RDW,%	17.5 (1.7)	17.9 (1.2)	**<0.001**^**b**^
MCHC, g/L	32.3 (2.0)	31.3 (2.7)	**<0.001**^**b**^

Data is presented as the median (interquartile range) unless stated otherwise. A total of 387 parasitemic children were categorized as either SMA (n = 165) or non-SMA (n = 222) based on age- and geographically-matched Hb concentrations in children in western Kenya (Hb < 6.0 g/dL, with any density parasitemia) [35]. ^a^Statistical significance determined by Chi-square (*χ*^2^) tests. ^b^Statistical significance determined by Mann–Whitney *U *test. Abbreviations: RBCs - Red blood cells; RDW - Red cell distribution width; MCHC - Mean corpuscular hemoglobin concentration.

### Distribution of individual IL-13 promoter variants in the clinical groups

The overall frequency of the -7402 T/G genotypes were TT (n = 334, 0.863), TG (n = 49, 0.127) and GG (n = 4, 0.010) (Table 2). There was no significant departure from Hardy Weinberg Equilibrium in the overall distribution (HWE, *χ*^2^ = 2.007, *P* = 0.157). The distribution of -7402 T/G variant in the non-SMA (n = 222) and SMA (n = 165) and the genotype frequencies within the clinical groups were TT (non-SMA, n = 194, 0.874; SMA, n = 140, 0.848), TG (non-SMA, n = 26, 0.117; SMA, n = 23, 0.139) and GG (non-SMA, n = 2, 0.009; SMA, n = 2, 0.012). These distributions were comparable between the SMA and non-SMA groups (*P* = 0.767). Additional analyses demonstrated that Hb levels were comparable across the genotypic groups (*P* = 0.744). There was no departure from HWE at the IL-13 -7402 T/G variant in either the non-SMA (*χ*^2^ = 1.104, *P* = 0.293) or SMA (*χ*^2^ = 0.861, *P* = 0.353) groups.

**Table 2:** The distribution of individual IL-13 promoter genotypes in the clinical groups

**Genotypes**	**Hemoglobin Levels (g/dL)**	***P *value**	**n (%)**	**Non-SMA (Hb ≥ 6.0 g/dL) n (%)**	**SMA (Hb < 6.0 g/dL) n (%)**	***P *value**
**IL-13 -7402 T > G**						
**TT**	6.4 (3.0)	0.744^b^	334 (86.3)	194 (87.4)	140(84.8)	0.767^a^
**TG**	6.1 (3.0)		49 (12.7)	26 (11.7)	23 (13.9)	
**GG**	6.2 (2.0)		4 (1.0)	2 (0.9)	2 (1.2)	
**IL-13 -4729 A > G**						
**GG**	6.4 (4.0)	0.521^b^	205(53.0)	78 (47.3)	127 (57.2)	0.152^a^
**GA**	6.0 (3.0)		131 (33.9)	63 (38.2)	68 (30.6)	
**AA**	6.2 (3.0)		51 (13.2)	24 (14.5)	27 (12.2)	

Data are presented as [n, (8722%)] for proportions of IL-13 promoter variants -7402 T > G and -4729 G > A within the SMA (n = 165) and non-SMA (n = 222) groups based on age- and geographically-matched Hb concentrations in children in western Kenya (Hb < 6.0 g/dL, with any density parasitemia) [35]. ^a^Chi-square analysis. The Hb levels are presented as medians (IQR), and ^b^Kruskal Wallis test was used to compare the levels between the genotypes.

The following were the overall genotypic frequencies at the -4729G/A locus; GG (n = 205, 0.530), GA (n = 131, 0.339), AA (n = 51, 0.132) (Table 2). Significant departure from the HWE was observed for this variant in the distribution of genotypes in the overall population (*χ*^2^ = 14.810, *P* < 0.001). Distributions of the genotypes within the clinical groups were GG (non-SMA, n = 78, 0.473; SMA, n = 127, 0.572), GA (non-SMA, n = 63, 0.382; SMA, n = 68, 0.306) and AA (non-SMA, n = 24, 0.145; SMA, n = 27, 0.122) and were comparable between the non-SMA and SMA groups (*P* = 0.152). In addition, the distribution of Hb levels were also comparable across the genotypic groups (*P* = 0.521). There was significant departure from HWE in the non-SMA group (*χ*^2^ = 11.891, *P* < 0.001) and marginal significant departure in the SMA group (*χ*^2^ = 3.458, *P* = 0.063).

### Association between individual IL-13 promoter polymorphisms and SMA

To determine the role of individual IL-13 genotypes in conditioning susceptibility to SMA, a multivariate logistic regression analyses were carried out using the wild type in each variant as the reference group, while controlling for the confounding effects of age, gender, bacteremia, G6PD deficiency and sickle-cell trait [10,13,36-38] (Table 3). Relative to the wild type genotypes (-7402, TT and -4729, GG), there was no significant association between the individual promoter variants and susceptibility to SMA at the -7402 T/G (TG, OR; 1.168, 95% CI, 0.626-2.180, *P* = 0.625 and GG, OR; 1.871, 95% CI, 0.240-14.597, *P* = 0.550) and -4729G/A (AA, OR; 1.484, 95% CI, 0.777-2.835, *P* = 0.232) loci. The GA genotype however showed marginal association with susceptibility to SMA (OR; 1.589, 95% CI, 0.995-2.539, *P* = 0.052).

**Table 3:** The associations between individual IL-13 promoter variants and SMA

**Genotypes**	**Odds Ratio**	**95% CI**	***P *value**
**IL-13 -7402 T > G**			
TT, (n = 334)	1.000	-	-
TG, (n = 49)	1.168	0.626-2.180	0.625
GG, (n = 4)	1.871	0.240-14.597	0.550
**IL-13 -4729 G > A**			
GG, (n = 205)	1.000	-	-
GA, (n = 131)	1.589	0.995-2.539	**0.052**
AA, (n = 51)	1.484	0.777-2.835	0.232

Multivariate logistic regression analyses were used to determine the odds ratio (OR) and 95% Confidence Interval (CI), controlling for age, gender, G6PD deficiency, sickle-cell trait status and presence of bacterial infections [10,13,36-38]. The homozygous wild-type genotypes were used as reference group for the regression analyses.

### Distribution of IL-13 promoter haplotypes in the clinical groups

Prior to determining the associations between the haplotypes and SMA, the overall distributions of the different haplotypes were determined. Results yielded the following overall distribution, -7402G/-4729G (GG, n = 53/387) were 13.7%, -7402 T/-4729A (TA, n = 181/387) were 46.8% and -7402 T/-4729G (TG, n = 311) were 80.4% (Table 4).

**Table 4:** The distributions of IL-13 promoter haplotypes in the clinical groups

**Haplotypes**	**Non-SMA (Hb ≥ 6.0 g/dL) n (%)**		**SMA (Hb < 6.0 g/dL) n (%)**	***P *value**
-7402G/-4729G	1	28 (12.6)	25 (15.2)	0.472^a^
	0	194 (87.4)	140 (84.8)	
-7402 T/-4729A	1	94 (42.3)	87 (52.7)	**0.043**^**a**^
	0	128 (57.7)	78 (47.3)	
-7402 T/-4729G	1	183 (82.4)	128 (77.6)	0.234^a^
	0	39 (17.6)	37 (22.4)	

Haplotypes were constructed based on the IL-13 variants using the HPlus software. On the Table, 1 represents carriers of the haplotypes, while 0 represents non-carriers. Distributions of haplotypes compared between the SMA and non-SMA groups using ^a^χ^2^ test.

The distribution of the haplotype carriers in the clinical groups were as follows (Table 4); GG (non-SMA, n = 28/222, 12.6%; SMA, n = 25/165, 15.2%) and TG (non-SMA, n = 183/222, 82.4%; SMA, n = 128/165, 77.6%). These distributions were not significantly different in the SMA (*P* = 0.472) and non-SMA (*P* = 0.234) groups. However, the frequency of the TA haplotype was significantly higher in the SMA (n = 87/165, 52.7%) relative to non-SMA (n = 94/222, 42.3%; *P* = 0.043) groups, implying that the TA haplotype could be modulating susceptibility to SMA.

### Association between IL-13 promoter haplotypes and SMA

A further multivariate logistic regression analyses, controlling for the potential confounders was used to determine the association between the IL-13 promoter haplotypes and SMA. The analyses (Table 5) revealed that relative to the non-TA group, the carriage of the TA (IL-13 -7402 T/-4729A) haplotype was associated with increased susceptibility to SMA (OR; 1.564, 95% CI, 1.023-2.389, *P* = 0.039). However, the other haplotypes, i.e. GG (*P* = 0.533) and TG (*P* = 0.307) did not alter susceptibility to SMA.

**Table 5:** The associations between IL-13 promoter haplotypes and SMA

**Haplotypes**	**Odds Ratio**	**95% CI**	***P *value**
-7402G/-4729G, (n = 53)	1.211	0.663-2.212	0.533
-7402 T/-4729A, (n = 181)	1.564	1.023-2.389	**0.039**
-7402 T/-4729G, (n = 311)	0.760	0.449-1.286	0.307

Multivariate logistic regression analyses were used to determine the odds ratio (OR) and 95% CI, controlling for age, gender, G6PD deficiency, sickle-cell trait status and presence of bacterial infections [10,13,36-38]. The haplotype non-carriers were used as reference groups for the analyses in the regression analyses.

### Functional relationship between IL-13 promoter variants and circulating IL-13 levels

In order to explore the relationship between the individual IL-13 genotypes and functional changes in IL-13 production, circulating IL-13 levels were compared across the different IL-13 genotypes. Results revealed that IL-13 levels were comparable across the -7402 T/G (*P* = 0.357) and -4729G/A (*P* = 0.438) genotypes. Further analyses demonstrated that the IL-13 haplotypes (GG; *P* = 0.662, TA; *P* = 0.830, TG; *P* = 0.240) did not alter changes in circulating IL-13 levels (data not presented).

## Discussion

To the best of our knowledge, this is the first study to report on the association between the IL-13 promoter polymorphisms (-7402 T/G and -4729G/A) and susceptibility to SMA in well-defined phenotypic pediatric populations resident in *P. falciparum* holoendemic transmission region of western Kenya. Our results, obtained from this cross-sectional study of a cohort of parasitemic children (n = 387) aged 3–36 months in this *P. falciparum* holoendemic region, revealed that none of the individual IL-13 genotypes was associated with susceptibility to SMA. Further analyses, however, revealed that the individuals with the -7402 T/-4729A (TA) haplotype were significantly over-represented in the SMA group relative to non-SMA. Carriage of the TA haplotype was associated with susceptibility to SMA, confirming our findings that this haplotype could confer genetic predisposition to SMA in children naturally exposed to *P. falciparum* infection. Frequencies of the TT genotype at the -7402 T/G genotypes was higher in our population relative to that of the reference Yoruba population [39], showing that this locus may be undergoing some selective pressure in our population either as a result of disease or any other unidentified selection. In addition, there is likely to be additional SNPs in linkage disequilibrium with the IL-13 variants that may in turn lead to the observed association between the studied SNPs and SMA. It would be important to map additional SNPs in the IL-13 loci and adjacent genes in order to further identify variants driven by disease-related selections.

Haplotypes are important markers for identifying associations with disease outcomes that are not identifiable with SNPs [11]. Previous studies, using different disease (other than SMA) models showed associations between individual (-7402 T/G and -4729G/A) and parasitic infections and allergic responses [25,30]. In the current study, carriage of the -7402 T/-4729A (TA) haplotype was associated with increased susceptibility to SMA. Consistent with the finding that TA haplotype is associated with susceptibility to SMA, the presence of the haplotype was over-represented in SMA relative to non-SMA group. Even though the mechanism through which carriage of the TA haplotype increases susceptibility to SMA remain to be determined in our laboratory, we hypothesize that the presence of the TA haplotype may be promoting non-erythropoietic responses in children with falciparum malaria through alteration of IL-13 promoter binding of transcription factors to the IL-13 promoter region. Previous studies have demonstrated that changes that affect IL-13 and IL-13Rα1 at the D1 domain of the type II IL-4 receptor [18], leading to alterations in signaling for production of IL-13 via activation of the transcription factor signal transducer and activator of transcription 6 (STAT6) may affect erythropoiesis [40]. Other studies have also shown that the IL-13Rα1 receptor forms a heterodimer with IL-4Rα which provide signaling pathways for both IL-13 and IL-4 [41-43]. IL-4 has further been shown to be an erythropoietic inhibitor whose elevated production can aggravate severe malaria [44]. As such, the presence of the TA haplotype could be altering the gene-gene interactions of IL-13 and IL-13Rα1, thereby promoting enhanced IL-4 production leading to inhibition of erythropoiesis in children with malaria. Since there were no relationships between individual IL-13 genotypes and haplotypes and circulating IL-13 levels in the current study, there is likely to be additional regulators either upstream or downstream of the IL-13 promoter that may alter the IL-13 signaling pathway during disease. Our laboratory is currently investigating the possibility of these additional regulators in functionally altering a milieu of immune mediators during malaria disease pathogenesis in children.

Previous studies have demonstrated that increased IL-13 production exacerbate infectious diseases and allergic disorders [25,30,45,46], alter immune responses in malaria [47-49], and is potentially a negative predictor of Hb levels in children with malarial disease [17]. Even though a previous study demonstrated that variants within the IL-13 (-1055C/T) promoter was associated with severe malaria in an adult Thai population [31], likely through alterations in the binding of transcription factor (NF-AT) on the IL-13 promoter region thereby leading to differential production of IL-13 levels, no study to date had determined the role of -7402 T/G and -4729G/A in altering IL-13 production in children with malaria. We demonstrate that these variants (genotypes and haplotypes) do not independently alter IL-13 circulating levels in our pediatric population. In addition, contrary to previous findings [25,47-49], circulating IL-13 levels did not significantly differ between the SMA and non-SMA in our cohort. Even though previous studies have additionally demonstrated that IL-13 is involved in alternative activation of macrophages, a mechanism specialized for defense against extracellular pathogens [50], it is important to note that *P. falciparum* is an obligatory intracellular pathogen [51], and thus, the defense mechanism provided by alternative activation of macrophages may not absolutely aid in defense against it.

Furthermore, differences in the current results versus previous findings [25,47-49] could be due to geographical and demographic differences which may concomitantly be accompanied by various selective pressure. For example, the current pediatric population is naturally exposed to additional conditions such as other parasitic infections, which may independently alter IL-13 production [52,53]. These other parasitic infections may (in the presence or absence of *P. falciparum* malaria) complicate IL-13 production in relation to other immune mediators [52,53]. It is also important to note that infectious diseases occur through complex and multifactorial mechanisms [10,22,36,47,53,54], and as such, in populations in which there is likely to be multiple co-infections, it would be critical to delineate and stratify them into those with clearly defined disease status and carry out a comprehensive analyses of an all inclusive immune mediator analyses to shed more light into those immune mediators that significantly alter malaria disease outcomes.

## Conclusion

The current study reports for the first time, the association between the IL-13 promoter polymorphisms and haplotypes (IL-13 -7402 T < G/ and -4729G/A) and SMA in parasitemic children below 3 years of age in a *P. falciparum* malaria holoendemic transmission area of western Kenya. Even though the IL-13 promoter genotypes and haplotypes did not independently alter circulating IL-13 levels, carriage of the -7402 T/-4729A (TA) haplotype appeared to be genetically predisposed to development of SMA once the children acquired *P. falciparum* malaria. It would be important to carry out additional studies in independent populations experiencing different malaria disease outcome(s) to validate these findings.

## Ethical approval

The study was approved by the Ethics Review Committee of the Kenya Medical Research Institute. Informed written consent was obtained from the parent or legal guardian of all children participating in the study.

## Competing interests

The authors declare that they have no competing interests.

## Authors’ contribution

WAO, EOM, ER, JMV, JMO, DJP and CO designed, carried out the survey studies in the rural population and participated in the drafting of the manuscript. WO and SBA performed the statistical analyses and participated in the drafting of the manuscript. All authors read and approved the final manuscript.

## Authors’ information

Department of Biomedical Sciences and Technology, Maseno University, Maseno, Kenya (WAO and EOM); Lecturer, Department of Medical Biochemistry, Maseno University, Maseno, Kenya (WO and SBO); Lecturer, Department of Medical Laboratory Sciences, School of Health Sciences, Masinde Muliro University of Science and Technology, Kakamega, Kenya (ER); Centre for Global Health Research, Kenya Medical Research Institute, Kisumu, Kenya (JMV); Research Officer, University of New Mexico/KEMRI Laboratories of Parasitic and Viral Diseases, Centre for Global Health Research, Kenya Medical Research Institute, Kisumu, Kenya (JMO); Professor and Director, Center for Global Health, Department of Internal Medicine, University of New Mexico, Health Sciences Centre, Albuquerque, NM, USA (DJP); Associate Professor, Department of Biomedical Sciences and Technology, and Director, Research, Publications and Consultancies, Maseno University, Maseno, Kenya (CO).
